# Long‐term misdiagnosis and neurologic outcomes of thallium poisoning: A case report and literature review

**DOI:** 10.1002/brb3.2032

**Published:** 2021-01-13

**Authors:** Hailing Liu, Geng Liao

**Affiliations:** ^1^ Department of Neurology Maoming People's Hospital Maoming China

**Keywords:** long‐term follow‐up, neuroelectrophysiology: toxicity, neurologic outcome, thallium poisoning

## Abstract

**Introduction:**

Thallium poisoning is a rare occurrence. Therefore, thallium poisoning is easily misdiagnosed and is often accompanied by a series of serious sequelae and can even result in death in severe cases. Here, we report long‐term follow‐up of a case of a patient who was poisoned with thallium on two separate occasions.

**Methods:**

A 43‐year‐old man was initially misdiagnosed as gastroenteritis, diabetic peripheral neuropathy, and Guillain–Barré Syndrome (GBS) within 21 months. The correct diagnosis was confirmed by blood and urine thallium assays. After Prussian blue treatment, thallium was undetectable in the blood by day 60. Following this investigation, a criminal suspect confessed to two instances of adulterating thallium sulfate in the patient's beverage. A 6‐year follow‐up was performed after discharge, and a comprehensive literature was review.

**Results:**

We found that the original gastrointestinal symptoms, skin lesions, and hair loss were reversed and had recovered, except for residual neurologic damage, even with long‐term rehabilitation.

**Discussion:**

Thallium intoxication may have been initially identified if neurologic symptoms had occurred concurrently with gastrointestinal and cutaneous symptoms. Neurologic damage represented the main sequelae of thallium poisoning in our present case report.

## Graphical abstract

Thallium poisoning; neurologic outcome; neuroelectrophysiology

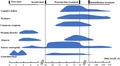

## 1 INTRODUCTION

Thallium is one of the most toxic heavy metals and is widely used in the manufacturing of highly regulated products (Baldwin, 1999). The hallmark symptoms of thallium poisoning are digestive issues, alopecia, and polyneuropathy, each of which present at different stages following poisoning (Galván‐Arzate, 1998). Here, we present findings from a patient who was poisoned with thallium on two separate occasions. We followed up the patient for 6 years to determine the long‐term outcomes of his thallium poisoning.

## 2 METHODS

This study has been performed in accordance with the ethical standards laid down in the 1964 Declaration of Helsinki. The Institutional ethics committee of Maoming people's Hospital had granted approval for our work.

We determined the detailed long‐term outcomes of a patient with two separate occasions of thallium poisoning. A PubMed literature review was performed using the terms “thallium poisoning” or “thallium intoxication.” Publications from January 1990 to September 2020 reporting more than two cases of thallium poisoning with detailed manifestations, treatments, and outcomes were selected. Demographics, intervals from thallium exposure to onset, clinical symptoms, determinations of thallium levels in urine, treatments, and outcome data were extracted from these selected studies.

## 3 RESULTS

### 3.1 Case details

**FIGURE 1:**
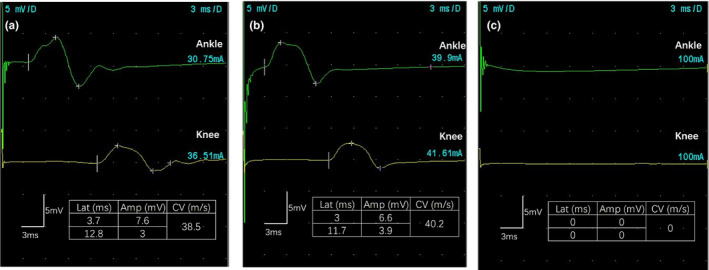
Neurophysiological evaluation of thallium poisoning at the onset of symptoms and at different follow‐up periods. Peripheral nerve conduction data showing reduced conduction velocity with mildly reduced compound muscle action potentials at the first onset of symptoms (a), which remained unchanged at the second onset of symptoms (b), but the evoked potential amplitude was absence, and the conduction velocity was nondetectable at a 2‐year follow‐up (c)

A 43‐year‐old man developed acute abdominal colic and diarrhea a few hours after a barbecue dinner. Gastroenteritis was diagnosed. Then, the patient continued to complain about the numbness of his limbs. His individual and family histories were unremarkable. Neurologic examination suggested hypoesthesia of the lower extremities. Blood glucose, urine glucose, and urine ketones were all positive. Nerve conduction studies demonstrated features of peripheral sensorimotor neuropathy (Figure 1a). Hence, a diagnosis of diabetic peripheral neuropathy was considered at this time. Following a combined treatment, the patient's symptoms gradually improved and he was subsequently discharged.

Twenty‐one months later, an aching pain in both distal lower limbs was experienced by the patient on one afternoon, which then progressed to extremely painful paresthesia, myalgia, and weakness in both of his legs. Alopecia started in the third week. Neurologic symptoms deteriorated continually in the fourth week. Thereafter, he was then transferred to another hospital. Guillain–Barré Syndrome (GBS) was diagnosed and treated. Unfortunately, the patient's specific symptoms were not improved at this time. Finally, the patient was transferred to the Department of Neurology at our hospital‐Maoming people's hospital.

Neurologic examination revealed that the patient was confused and exhibited incoherent speech, dysesthesia, and a symmetrical decrease in muscle strength of the lower extremities (4/5 in proximal muscles and 0/5 in distal muscles), along with an absence of tendon reflexes. The patient's score on the Mini‐Mental State Examination (MMSE) was 13, and his Barthel index (BI) was 20. Results of routine laboratory tests were all normal. Electrocardiographic data indicated tachycardia. Nerve conduction studies demonstrated features of peripheral sensorimotor neuropathy, similar to the findings during the aforementioned first onset of symptoms (Figure 1b). Results of brain magnetic resonance imaging were normal.

Considering the presence of both severe peripheral neuropathies and alopecia, heavy metal intoxication was suspected. Testing for arsenic, mercury, and lead were all negative. Blood and urinary thallium concentrations of the patient were determined by atomic absorption spectrophotometry, and they were all significantly elevated compared with those of normal levels. Thallium levels were 336.5 μg/L in the patient's blood (normal < 10 μg/L) and 252.3 μg/L in the patient's urine (normal < 20 μg/L). Hence, the diagnosis of thallium poisoning was confirmed. Prussian blue (250 mg/kg/d in 4 divided doses) was administered for 2 months until blood thallium concentrations returned to normal levels. Following treatment, the patient's symptoms were alleviated significantly, although lower extremity paralysis, numbness, and mild cognitive impairment were still present (MMSE 19, BI 20). The patient was then transferred to our rehabilitation department for long‐term rehabilitation treatment.

At a 6‐month follow‐up after treatment, the clinical cutaneous features had subsided completely. Considering that the patient had diabetes mellitus, blood sugar levels remained normal upon treatment with insulin. Muscle strengths of the patient's legs were graded 4/5 in proximal muscles and 1/5 in distal muscles, and the MMSE and BI scores improved to 23 and 20, respectively.

At a 2‐year follow‐up, there was residual mild weakness and numbness of the distal lower extremities. Muscle strength was graded 3/5 in distal muscles. The MMSE and BI scores were 29 and 75, respectively. The signs of electrophysiology aggravated comparing to previous investigations. Main findings of nerve conduction showed the evoked potential amplitude was absence, and the conduction velocity was nondetectable at a 2‐year follow‐up (Figure 1c). At a 6‐year follow‐up, the record muscle strengths and MMSE and BI scores did not exhibit any improvements compared with those at the previous follow‐up. The detailed scores at different time points are listed in Table 1.

**TABLE 1:** Muscle strength (MS), Mini‐Mental State Examination (MMSE), and Barthel index (BI) in a patient with thallium poisoning at different time points

Time	First onset	Second onset	After treatment	6‐month follow‐up	1‐year follow‐up	2‐year follow‐up	6‐year follow‐up
MS	5	0	1	1	2	3	3
MMSE	30	13	19	23	24	29	29
BI	100	15	20	20	35	75	75

**FIGURE 2:**
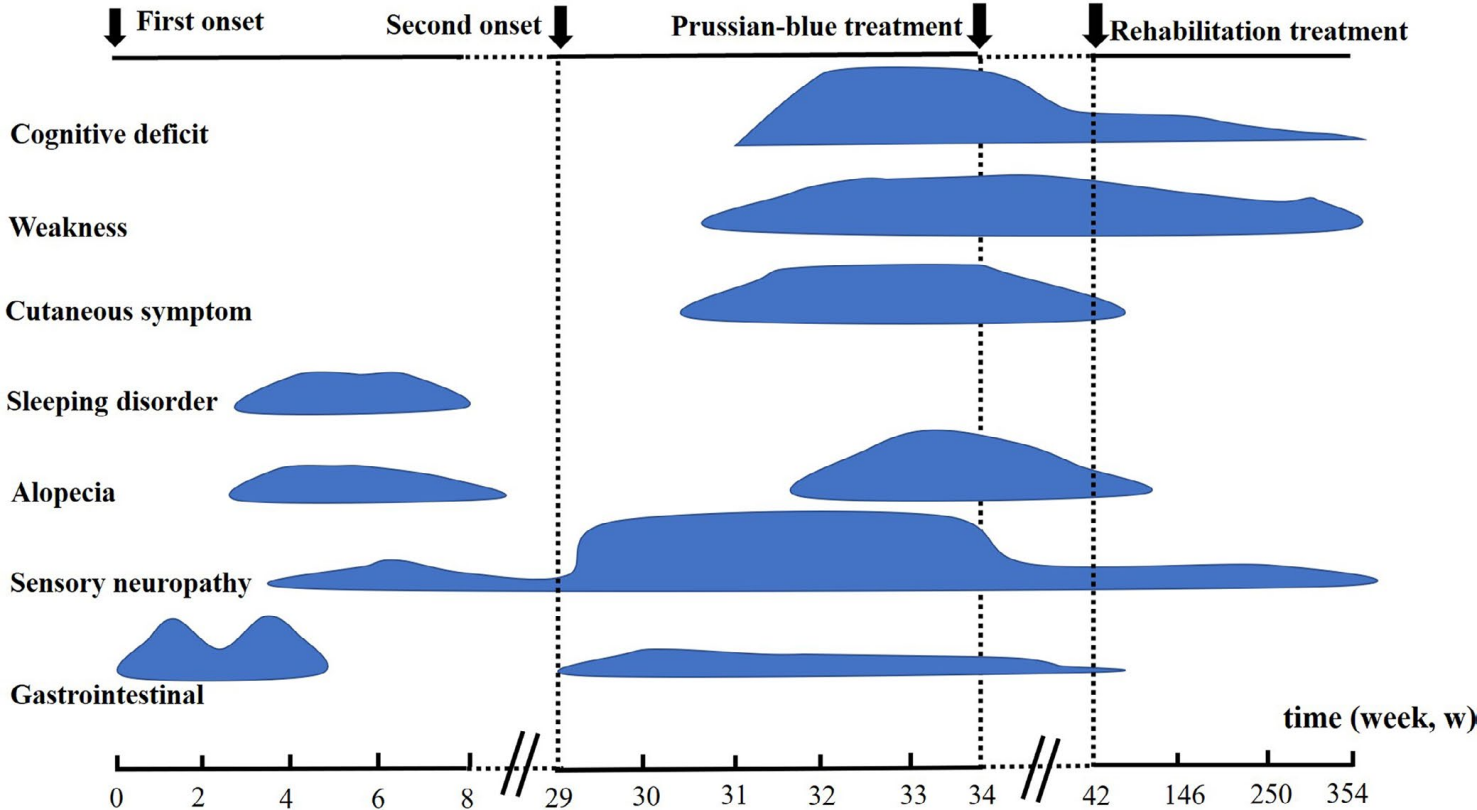
The relationship between two incidents of thallium poisoning and variation in the main clinical manifestations from the first onset to a 6‐year follow‐up

Since the patient was suspected of being poisoned, an epidemiological study was conducted to investigate his work and home environments. Following this investigation, a criminal suspect confessed to two instances of adulterating thallium sulfate in the patient's beverage, although the dosages of thallium sulfate were unable to be determined. The relationship between the two incidents of thallium poisoning and variation in the main clinical manifestations from the first onset to 6 years into follow‐ups is shown in Figure 2.

### 3.2 Literature review

Nine articles including 98 cases with thallium poisoning were retrieved in our literature review (Table 2) (Afshari et al., 2012; Al Hammouri et al., 2011; Desenclos et al., 1992; Di Candia et al., 2020; Li et al., 2014; Lin et al., 2019; Meggs et al., 1994; Sun et al., 2012; Zhang et al., 2014). The median age of the patients was 30 years (range 2–73 years). The number of intervening days from exposure to onset was 1–6 days. The clinical characteristics of these patients were systematically analyzed and are presented in Table 2. We found that polyneuropathy (82%), alopecia (68%), and abdominal pain (51%) were the most frequent clinical manifestations, which is consistent with a previous study (Baldwin & Marshall, 1999). Determination of thallium in urine ranged from 68 to 42,000 μg/L. The treatments included symptomatic treatment, prussian blue alone, or combined with hemoperfusion, hemodialysis, plasma exchange, or continuous renal replacement therapy (CRRT). In terms of outcomes, we determined that 55% of patients had subsided symptoms, 12% died, and 20% of patients remained with neurologic sequelae. However, statistical bias may have existed due to missing data.

**TABLE 2:** Systematic review of cases of thallium poisoning

References.	Afshari et al., 2012	Desenclos et al., 1992	Di Candia et al., 2020	Li, et al., 2014	Lin et al., 2019	Meggs et al., 1994	Sun et al., 2012	Zhang et al., 2014	Al Hammouri et al., 2011	Total
Numbers	3	3	8	13	34	4	14	9	10	98
Age (year)	26 (22–32)	24 (16–41)	—	37 (15–53)	39 (26–53)	—	36 (2–73)	—	18 (2–42)	30 (2–73)
Interval from exposure to onset (d)	—	2 (1–3)	—	2.3 (1–6)	—	2.5 (1–3)	—	2 (1–3)	2 (1–3)	2 (1–6)
Symptoms										
Gastrointestinal										
Nausea and vomiting	0	1	8	—	1	3	7	6	8	34 (34%)
Abdominal pain	0	3	8	—	12	3	11	6	8	51 (52%)
Abdominal distension	0	1	8	—	9	—	6	4	8	36 (36%)
Neurologic										
Polyneuropathy	1	3	8	12	31	4	14	2	7	82 (83%)
Tremor or weakness in extremities	3	3	8	8	1	—	7	2	8	40 (40%)
Dizziness and headaches	0	0	—	3	—	—	6	3	7	19 (19%)
Vision loss	3	0	—	—	—	—	—	—	3	6 (6%)
Delirium	0	2	—	5	—	—	3	1	5	16 (16%)
Coma	0	2	—	1	1	—	1	—	6	11 (11%)
Cardiac arrhythmias	0	2	—	3	—	2	—	1	—	8 (8%)
Dyspnea	—	3	3	—	—	—	—	1	—	7 (7%)
Dermatological										
Alopecia	3	3	—	10	19	3	14	9	7	68 (69%)
Mees' lines	0	—	—	3	—	—	—	2	1	6 (6%)
Erythematous skin rashes	0	—	—	2	—	—	2	1	4	9 (9%)
Fatigue	3	—	8	—	—	—	14	9	—	34 (34%)
Insomnia	1	—	—	3	—	1	—	—	9	14 (14%)
Thallium in Urine (μg/L)	2,000–3,000	68–26,000	16,300–42,000	231–12,840	—	1,083.7–9,569	956.5–11,285	4,343.5–9,998	452–2,909	68–42,000
Treatments	Not PB nor PF	—	PB (8)	PB (8); PB + HP (5)	PB (16); PB + HP (12)/PE (1)/CRRT (1)	PB + HD (4)	PB (2) PB + HD (12)	PB + HP/HD/CRRT (9)	PB (1); PB + HD (9)	—
Outcome										
Neurologic sequelae	1	2	—	11	5	0	1	0	—	20 (20%)
Dead	0	1	3	2	0	0	0	0	3	9 (12%)
Complete reversal	2	0	—	—	26	4	13	9	—	54 (55%)

Abbreviations: −, means not available; CRRT, continuous renal replacement therapy; HD, hemodialysis; HP, hemoperfusion; PB, Prussian blue; PE, plasma exchange.

## 4 DISCUSSION

Thallium poisoning most often occurs after accidental poisoning or an attempted homicide (Rodríguez‐Mercado and Altamirano‐Lozano, 2013). The primary route of poisoning is via ingestion, although rare cases of skin and respiratory absorption have also been reported. The mechanisms of thallium toxicity involve the substitution of potassium in Na‐K ATPases, because thallium closely resembles potassium in terms of its ionic size and charge, and thallium also interacts with sulphydryl groups of molecules in mitochondrial membranes and neuronal axons (Baldwin, 1999).

A comprehensive literature search from PubMed identified nine studies of three or more cases of thallium poisoning each. In these cases, the durations from symptom onset to diagnosis ranged from several hours to four years, even after autopsy (Hirata et al., 1998; Li et al., 2014), and this wide range may be due to the following factors. Characteristics of thallium poisoning are attributed to the corresponding dose, as well as individual susceptibility and metabolism. In previous studies (Herrero et al., 1995), thallium has been shown to affect multiple organs and to induce several clinical symptoms, including gastrointestinal, neurologic, dermatological, circulatory, and respiratory symptoms. Gastrointestinal symptoms presenting with nausea, vomiting, abdominal pain, and distension are usually the first characteristics of thallium poisoning and can be easily ignored. Neurologic damage following thallium poisoning can include polyneuropathy (presenting with pinprick pain), paresthesia, dysesthesia, and/or hypoalgesia in the distal lower limbs. About 2–3 weeks later, characteristics of dermatological changes can include alopecia, Mees' lines, and/or erythematous skin rashes. In addition, thallium poising is often accompanied by nonspecific symptoms, such as headaches, myalgia, depression, and sleeping disturbances. Results of routine laboratory tests are usually unremarkable, although hepatic/renal damage, abnormal full blood counts, hyponatremia, hypokalemia, and/or high blood glucose may be detected during the initial stage of severe cases (Zhu et al., 2019). In our present case, the evidence that the patient presented high blood glucose may have been a coincidence due to him being diabetic and without recent exogenous insulin at the time of the follow‐up.

Although the distinctive clinical features of thallium poisoning comprise gastrointestinal presentation, alopecia, and polyneuropathy (Galván‐Arzate, 1998), these features are not always present initially during clinical examinations, which can lead to misdiagnoses. In previous cases (Baldwin & Marshall, 1999), thallium poisoning has often been misdiagnosed as gastroenteritis, peripheral neuropathy, and GBS at the early stages of symptoms.

In our present case report, retrospective analysis showed that the patient's symptoms were typical of thallium poisoning. Therefore, a lack of experience of the attending physician who initially assessed this patient may have played an important role in the initial misdiagnosis.

Thallium intoxication can severely affect the nervous system, including peripheral (sensorimotor neuropathy), central (altered mental status, convulsions, and extrapyramidal and cerebellar dysfunctions), and autonomic (tachycardia, hypertension, and urinary retention) neural systems. At considerably high thallium doses, coma and death may occur (Tsai et al., 2006). A previous study reported a case of neurologic symptoms that rapidly and severely deteriorated after the second thallium ingestion, but that did not exhibit any other symptoms (Kuroda et al., 2016). The long‐term prognosis of thallium poisoning depends on the total dose ingested, the timing of treatment, and the individual susceptibility of each patient (Huang et al., 2014). Most of the symptoms of thallium poisoning can be completely reversed, but mild or moderate neurologic sequelae may last for a long time. In the outcome of review studies, we found that 55% patients recovered, 12% died, and 20% patients remained with neurologic sequelae presenting with paresthesia, weakness in the extremities, and/or cognitive dysfunction. In the present case report, after treatment with Prussian blue and long‐term rehabilitation, at a 6‐year follow‐up, the patient's muscle strength in the distal muscles of his legs improved (from 0 to 3), as did his BI (from 15 to 75). Nonetheless, neuroelectrophysiological signatures were not improved at this follow‐up. Nerve conduction was undetected 2 years later, and electromyographic data were suggestive of axonal degeneration. In a previous study of a patient with thallium poisoning, sequential nerve conductions revealed that nerve conduction was undetected 2 years after initial symptoms, despite the patient otherwise improving clinically (Dumitru & Kalantri, 1990). This finding suggests the possibility of clinical improvements following thallium poisoning, despite continued deterioration of electromyographic signatures. The prominent central neurologic symptoms of our present case report—which included confusion, psychosis, and cognitive deficits—improved significantly at follow‐ups. MMSE scores gradually increased from 13 to 29, but subtle cognitive deficits persisted even at a 6‐year follow‐up.

## 5 CONCLUSIONS

In our present case report, thallium intoxication may have been initially identified if neurologic symptoms had occurred concurrently with gastrointestinal and cutaneous symptoms. Neurologic damage represented the main sequelae of thallium poisoning in our present case report.

## CONFLICT OF INTEREST

None of the authors has any conflict of interest to disclose.

## AUTHOR CONTRIBUTIONS

We declare that all the listed authors have participated actively in the report. GL designed the report and wrote the protocol. HL wrote the first draft of the manuscript. GL and HL managed the literature searches and analyses. GL and HL took overall responsibility.

## ETHICAL APPROVAL

We confirm that we have read the Journal's position on issues involved in ethical publication and affirm that this report is consistent with those guidelines.

### Peer Review

The peer review history for this article is available at https://publons.com/publon/10.1002/brb3.2032.

## Data Availability

The datasets used or analyzed during the current study are available from the corresponding author on reasonable request.
